# Editorial: The role of calcium signaling in gametogenesis and early embryogenesis

**DOI:** 10.3389/fcell.2022.1051435

**Published:** 2022-10-11

**Authors:** Junaid Kashir, F. Anthony Lai, Michail Nomikos

**Affiliations:** ^1^ Department of Biology, College of Arts and Sciences, Khalifa University, Abu Dhabi, United Arab Emirates; ^2^ Department of Comparative Medicine, King Faisal Specialist Hospital and Research Centre, Riyadh, Saudi Arabia; ^3^ College of Medicine, Qatar University, QU Health, Doha, Qatar

**Keywords:** calcium, reproduction, fertilisation, embryogenesis, sperm, oocyte, gametogenesis

Calcium homeostasis exerts a profound role in determining the quality of gametogenesis, the resultant efficacy of fertilisation and ultimately the competency of embryogenesis. Perhaps no other single ion plays such a profound and determinative role in reproductive success as calcium, since it is critical for germinal vesicle breakdown, sperm capacitation and hyperactivation, oocyte activation, maintenance of zygote calcium homeostasis and embryogenic developmental gradients.

This Research Topic aimed to collect, collate, and summarize the most recent and cutting-edge advances and propositions studying the molecular determinants and effectors of calcium signalling and homeostasis throughout the early events of fertilisation and preimplantation embryogenesis, with a specific view to examine how calcium can affect the efficacy of such important phenomena.

This Research Topic includes two novel reviews and a perspective. Shum et al. summarise the mechanisms that regulate calcium homeostasis in the epididymis, an essential organ for sperm maturation, wherein sperm cells are protected and acquire mobility and the ability to fertilize. More specifically, this review focusses on the potential role of vitamin interactions on epididymal calcium, especially the role of calcium in the epididymal lumen as a cofactor for the matrix Gla protein (MGP), which plays an essential role in promoting calcium-dependent protein aggregation. Alhajeri et al. examine the potential connection between neuronal signaling and physiological changes during oocyte maturation and fertilization, providing an overview of reported neurotransmitters and neuropeptides that participate in early embryogenesis in relation to calcium signaling. Shafqat et al., in their novel perspective article, examine the potential links between alterations in cytosolic calcium within the fertilising zygote in the context of the dynamics of DNA methylation during this process. The authors draw upon cancer research that has examined such links in more detail compared to reproduction.

Three additional original articles complete this edition. In their brief research report, Savy et al. examine oocyte calcium in the context of superovulation, a common approach utilising extra-physiological levels of gonadotropins to promote continued ovarian follicle maturation that otherwise would undergo atresia, to ultimately maximize the number of oocytes available for either clinical assisted reproductive technologies or experimental animal studies. As evidence suggested a detrimental effect of superovulation upon the quality of oocytes/embryos, the authors examined the hypothesis that this procedure resulted in a diminished capacity for calcium release at fertilisation. The authors find that despite subtle differences in calcium patterns, superovulation did not disrupt physiological calcium signalling at fertilization, and supported the continued use of this method for both clinical and experimental purposes.

Meng et al. examine the potential role of calcium within *in vitro* maturation (IVM) and oocyte reprogramming in low calcium culture conditions. The authors observed a delayed rate of first polar body extrusion, and a delayed mitochondrial and endoplasmic reticulum organisation, suggesting important roles for calcium within oocyte maturation. Indeed, this low-calcium derived defective maturation seemed to also underlie poor developmental profiles of somatic cell nuclear transfer (SCNT) embryos. It is also proposed that low calcium may promote oxidative stress and apoptosis at both maturation and early embryogenesis.

Chen et al. examine the role of the *SEPTIN12* gene in the context of male infertility, identifying a novel heterozygous mutation within the *SEPTIN12* gene of the male partner in an infertile couple with a history of fertilisation failure (a process usually linked to poor calcium dynamics). Indeed, this couple was able to achieve successful fertilisation and pregnancy following intracytoplasmic sperm injection (ICSI) with artificial oocyte activation (AOA; eliciting calcium release via artificial compounds). The authors also generated a *Septin12* knockout (KO) mouse model finding that only homozygous KO mice were infertile, exhibiting diminished sperm counts and abnormal sperm morphology. Rather intriguingly, not only did AOA rescue the failed fertilisation phenotype of homozygous KO mice, but also enhanced the rate of embryogenesis progression to the 2-cell stage, suggesting an improvement in embryogenesis quality. The authors demonstrated a reduced level of phospholipase C zeta (PLCζ; the mammalian sperm factor thought responsible for eliciting calcium release at fertilisation) within *Septin12* homozygous KO sperm, linking *Septin12* to PLCζ and oocyte activation probably via mediation of sperm quality.

Collectively, we hope that this article collection further enhances our understanding of the demonstrated and theorised roles played by calcium in almost all aspects of reproduction, all the way from gametogenesis to fertilisation and finally embryogenesis. We anticipate that these articles will be of significant value to the community of reproductive medicine and biology, and help pave the way for further understanding the complexity and enormity of calcium signalling in mammalian reproduction.

